# The Assembled and Annotated Genome of the Fairy-Ring Fungus *Marasmius oreades*

**DOI:** 10.1093/gbe/evab126

**Published:** 2021-05-29

**Authors:** Markus Hiltunen, Sandra Lorena Ament-Velásquez, Hanna Johannesson

**Affiliations:** Department of Organismal Biology, Uppsala University, Sweden

**Keywords:** mushroom, repeat library, transposon, linkage map, genomics, Agaricomycetes

## Abstract

*Marasmius oreades* is a basidiomycete fungus that grows in so called “fairy rings,” which are circular, underground mycelia common in lawns across temperate areas of the world. Fairy rings can be thought of as natural, long-term evolutionary experiments. As each ring has a common origin and expands radially outwards over many years, different sectors will independently accumulate mutations during growth. The genotype can be followed to the next generation, as mushrooms producing the sexual spores are formed seasonally at the edge of the ring. Here, we present new genomic data from 95 single-spore isolates of the species, which we used to construct a genetic linkage map and an updated version of the genome assembly. The 44-Mb assembly was anchored to 11 linkage groups, producing chromosome-length scaffolds. Gene annotation revealed 13,891 genes, 55% of which contained a pfam domain. The repetitive fraction of the genome was 22%, and dominated by retrotransposons and DNA elements of the KDZ and *Plavaka* groups. The level of assembly contiguity we present is so far rare in mushroom-forming fungi, and we expect studies of genomics, transposons, phylogenetics, and evolution to be facilitated by the data we present here of the iconic fairy-ring mushroom.

Significance

Genome assemblies of mushroom-forming fungi with complete chromosome sequences are currently rare, and the large clade of *Marasmius* is particularly undersampled, obstructing studies of genome evolution in this part of the fungal tree of life. Here, the nearly gapless chromosome and mitochondrion sequences of *Marasmius oreades*, commonly known as the Scotch bonnet or fairy-ring mushroom, are presented, alongside a high-quality gene annotation and repeat library. These data resources open up the opportunity to answer detailed questions about mushroom evolution.

## Introduction

**Fig. 1.**
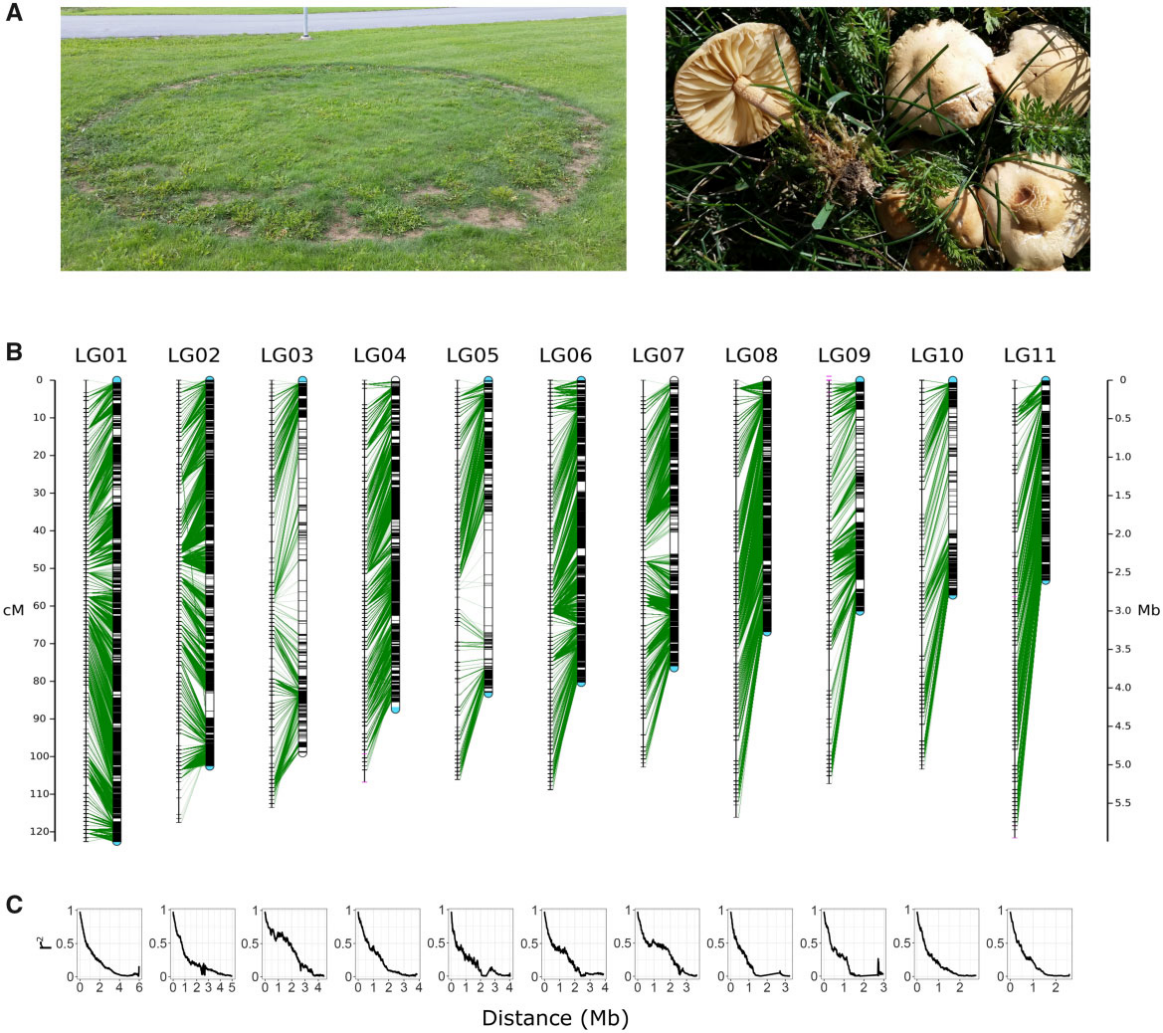
Linkage in the *Marasmius oreades* genome. (*A*) Photographs of a *M. oreades* fairy ring (˚ 6.7 m) and fruiting bodies (photos by M. Hiltunen). (*B*) The 11 linkage groups (LGs) and corresponding putative chromosome sequences of the *M. oreades* genome. The LGs are shown as lines to the left, with marker positions in cM distance drawn as horizontal lines across the LGs. The corresponding chromosome sequences are show to the right, with green lines connecting the marker genetic and physical coordinates. Markers mapping to another chromosome than the expected one are shown in magenta on the LGs (e.g., at the end of LG04). Telomere repeats are indicated in blue on the chromosome sequences (not drawn to scale). (*C*) Linkage disequilibrium (*r*^2^) decay between markers with increasing physical distance for each linkage group.

*Marasmius oreades* is a member of the ubiquitous and important Marasmiaceae, a family of mainly litter-decaying basidiomycete fungi. The species, commonly known as the edible Scotch bonnet, is known for forming fairy rings; long-lived circular, underground mycelia that are commonly found in lawns and grasslands (fig. 1*A*). This characteristic makes the species a suitable model for evolutionary studies, as individual mycelia are clearly discernable and the age of the rings can be inferred from the diameter (Burnett and Evans 1966). Fairy rings can be thought of as natural evolution experiments, where different sectors of the rings are subject to evolutionary forces such as mutation and selection during an extensive vegetative growth phase (Hiltunen et al. 2019). Mushrooms develop seasonally along the edge of the ring, where the sexual progeny in the form of basidiospores is produced, enabling the study of inheritance of variants present in the ring. The genome assembly of *M. oreades* was recently presented and used to study primarily single-basepair mutations accumulating during growth (Hiltunen et al. 2019). However, mutations affecting larger chromosomal regions, including movement of transposable elements (TEs), are increasingly being recognized as important additional sources of variability in natural populations (Barrón et al. 2014; Stuart et al. 2016; Oggenfuss et al. 2020). An improved version of the *M. oreades* genome assembly, with nearly completely assembled chromosomes, is expected to enable studies of such mutations in this system. Fungi typically have small genome sizes compared with other eukaryotes. Hence, whole-genome sequencing of haploid offspring can be used cost-effectively to produce linkage data of different chromosomal regions (Labbé et al. 2008; Foulongne-Oriol 2012; Fierst 2015).

## Results and Discussion

**Fig. 2.**
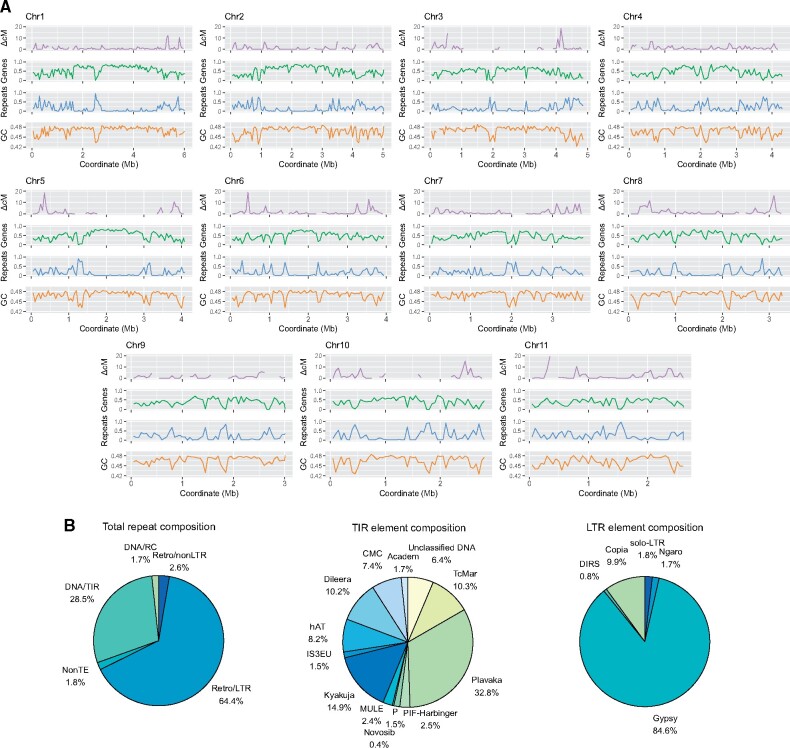
Genome characteristics. (*A*) Panels from top to bottom: recombination rate as difference in mean cM distance between windows; gene density as fraction of occupied base pairs per window; repeat density as fraction of occupied base pairs per window; GC content as fraction of bases per window. In all tracks, 50-kb nonoverlapping windows were used. (*B*) Repeat content in the genome as total repeat composition, and composition of the two most abundant classes: TIR and LTR elements.

In this study, we present genomic data of 95 single-spore isolates of *M. oreades* (supplementary table S1, Supplementary Material online)*.* These data are a valuable resource to study heritage and meiotic processes, and were here used to construct a genetic linkage map of the species. The linkage map consisted of 11 high-confidence linkage groups (LGs), spanning a total distance of 1,226 cM and a mean of 111 cM per LG (fig. 1*B*). We used the linkage map as a basis for a new genome assembly by reassembling previously sequenced PacBio, Oxford Nanopore MinION (ONT) long reads, and 10X Genomics (10XG) Chromium linked reads (supplementary table S1, Supplementary Material online) (data released in Hiltunen et al. [2019])*.* We were able to anchor 98% of assembled sequence (43,635,136 out of 44,372,355 bp) to the 11 LGs of the map, producing scaffolds of putative chromosome length (supplementary table S2, Supplementary Material online). The assembly size of *M. oreades* is smaller than that of the related *Marasmius fiardii* (59,447,912 bp) (Miyauchi et al. 2020). This difference may be explained by a true difference in genome size between the species and/or that the *M. fiardii* assembly is partly heterozygous as a result of sequencing a dikaryotic culture. As expected, linkage disequilibrium in the *M. oreades* genome degrades over physical distance between markers on the chromosomes (fig. 1*C*). Twenty-two gaps remain in the genome (lengths unknown). Of the assembled scaffolds, seven were found to start and end in telomere repeat sequence (TAAC[3-8], or the reverse complement), with the remaining four containing either the 5′ or 3′ telomere repeat (fig. 1*C*). Linked read data do not support linkage between the scaffolds with only one assembled telomere, causing us to believe that the telomeres in those cases are in reality close to the scaffold ends but were not anchored or assembled. Coverage of the spore isolates, and fractions of shared 10X Chromium barcodes between adjacent regions are mostly even across the scaffolds (supplementary fig. S1, Supplementary Material online). The recombination rate appears to increase towards the subtelomeres (fig. 2*A*). Although the exact determination of centromere regions would require additional experimental data, the combination of low recombination rate, low gene, and high repeat density, in addition to a drop in GC content may indicate such regions in our assembly (e.g., at ∼2.5 Mb on Chr1; fig. 2*A*). The mitochondrial genome was assembled to a 66,224 bp contig; a typical size for Agaricomycetes (Araújo et al. 2021). Twenty-one scaffolds (737,219 bp) remained unanchored, one of which contained a telomere repeat. No bacterial contamination was detected (supplementary fig. S2, Supplementary Material online). Almost all expected single-copy conserved orthologs (BUSCOs) could be found in the genome (98.5% complete + fragmented orthologs; basidiomycota_odb9, *n* = 1,335), confirming its high base-level accuracy (Simão et al. 2015). The level of assembly contiguity presented here is crucial for the study of, for example, TEs and chromosome evolution (Thomma et al. 2016), and yet, is rare among mushroom-forming fungi: less than 8% of available Agaricomycotina genomes on JGI Mycocosm (*n* = 467; https://mycocosm.jgi.doe.gov/mycocosm/home; accessed January 26, 2021) have L95 values of 20 or lower (most studied fungi in the Agaricomycotina have between 10 and 20 chromosomes).

Our *M. oreades* genome assembly was subjected to annotation of genes and repetitive sequences. The analysis predicted 13,891 genes (15,053 mRNAs), covering 55.5% (24,614,704 bp) of the genome, with an average gene length of 1,772 bp and six exons per mRNA (supplementary table S2, Supplementary Material online); estimates comparable to other Agaricomycetes (Ohm et al. 2010; Li et al. 2018; Miyauchi et al. 2020). The predicted protein set showed high concordance with the basidiomycete conserved ortholog database (98.5% complete + fragmented BUSCOs; basidiomycota_odb9, *n* = 1,335). Out of the predicted mRNAs, 8,222 (55%) contained a pfam domain (supplementary table S2, Supplementary Material online). Genes are generally concentrated towards the center of chromosomes and decrease in density towards the telomeres (fig. 2*A*). For confident TE calling, we constructed a library of repetitive sequences de novo, and manually curated the sequences therein (supplementary table S3, Supplementary Material online). Repeat annotation revealed 21.8% (9,685,063 bp) of the genome to be repetitive, and repeats tend to form regional clusters on the chromosomes (fig. 2*A*), similarly to in other fungi (Stajich et al. 2010; Foulongne-Oriol et al. 2013). Long-terminal repeat (LTR) elements are the most abundant mobile elements, covering 14% of the genome (fig. 2*B* and supplementary table S2, Supplementary Material online), which reflects findings in other basidiomycetes (Labbé et al. 2012; Amselem et al. 2015; Castanera et al. 2016). DNA elements constitute 6% of the genome, about half of which belong to the KDZ or *Plavaka* groups (Iyer et al. 2014). We characterized the terminal inverted repeat (TIR) motif of these understudied elements and summarized the information in supplementary figure S3, Supplementary Material online.

Taken together, the data presented here enable the use of *M. oreades* as a model for detailed studies of, for example, structural mutations, TE mobilization, and gene family evolution.

## Materials and Methods

Here, we generated genomic data of 95 single-spore isolates of *M. oreades*. Spore prints were collected from four fruiting bodies from one fairy ring located in a lawn in Uppsala, Sweden (59°51′27.3″N, 17°34′19.2″E). Spores were germinated on culture plates containing potato dextrose agar, and newly germinated single spores were transferred to fresh plates. Successful isolation of single-spore cultures was verified by inspecting cultures for absence of clamp connections, which is diagnostic of the monokaryotic (haploid) condition in this and many other species (Mallett and Harrison 1988). Isolates were transferred and grown in 2% liquid malt extract for several days before DNA purification with the Zymo Fungal/Bacterial Miniprep kit. Paired-end libraries (2 × 151 bp) were prepared from 1 ng DNA with the Nextera XT kit with dual indexes (Illumina) for each isolate separately, before pooling equal volumes of all 95 libraries and sequencing on one lane of Illumina HiSeqX (HiSeq X SBS chemistry).

For the genome assembly and annotation, we utilized published PacBio, ONT, and 10XG genome sequence reads, and Illumina HiSeq transcriptome reads (supplementary table S1, Supplementary Material online) (Hiltunen et al. 2019). In short, these data were collected the following way: a monokaryotic single spore of the species was isolated (strain 03SP1), grown in liquid malt extract, and DNA was obtained by the Qiagen Genomic Tip. A linked-read library was prepared from this DNA (10X Genomics Chromium Genome) and sequenced (half a lane of HiSeqX). For PacBio and Nanopore sequencing, the DNA was further purified using the MoBio PowerClean kit. For PacBio, a 10-kb insert size library was prepared and sequenced with RSII (eight SMRT cells, C4 chemistry, P6 polymerase). Two Nanopore libraries were prepared, one with the LSK108 kit and sequenced on a R9.4 flowcell, and another with the LSK308 kit, sequenced on a R9.5 flowcell. A cDNA library was prepared, using the same culturing method as above, but immediately freezing harvested tissue in liquid nitrogen, extracting total RNA with the RNeasy Plant Mini kit (QIAGEN, Germany), preparing the library with NEBNext Ultra Directional RNA Library Prep kit for Illumina (New England Biolabs). Sequencing was performed in one lane of HiSeq2500 (v4 chemistry).

We newly assembled the data presented by Hiltunen et al. (2019) as follows. Raw PacBio and Nanopore reads were assembled using Canu v1.7 (Koren et al. 2017). This backbone assembly was split at misassembly positions as identified by Tigmint v1.1.2 (Jackman et al. 2018) from a BWA mem 0.7.17-r1188 (Li and Durbin 2009) mapping of the 10XG reads. Scaffolds were built by ARBitR v0.1 (Hiltunen et al. 2020) using the 10XG reads mapped by EMA v0.6.2 (Shajii et al. 2018) to the broken assembly. Scaffold gaps were filled using LR_Gapcloser (Xu et al. 2019) and error-corrected long reads from Canu. Next, reasoning that the gap filling step may have introduced some sequence that was already present in contigs that could not be integrated into scaffolds, the assembly was scanned for such redundant contigs, by splitting the assembly into long and short scaffolds (threshold: 100 kb) and aligning the short ones to the long ones with the nucmer program of MUMmer v4.0.0beta2 (Kurtz et al. 2004). Short contigs with >97% sequence identity and >95% alignment coverage to a long scaffold were removed. The resulting assembly was subjected to another round of ARBitR scaffolding, gap filling, and removal of redundant contigs as above. Then the next step was to create a genetic linkage map to anchor and order the assembled scaffolds into long pseudomolecule sequences.

After adapter and primer trimming of the 95 single-spore isolate sequencing reads using Trimmomatic v0.32 (Bolger et al. 2014), reads were mapped to the scaffolded assembly using BWA mem. We used Lep-MAP3 v0.2 (Rastas 2017) to generate a linkage map from these data, by calling variants using Samtools mpileup (Li et al. 2009) and piping into Lep-MAP3. Variants were filtered with the Lep-MAP3 module Filtering2; allowing for a heterozygote rate of 0.1 (in theory, no sites can be heterozygous in the haploid spore isolates, but we included this parameter due to index hopping; van der Valk et al. 2020), a maximum of ten individuals with missing data per site, and noninformative sites removed. After filtering, 141,011 sites remained. A linkage map was constructed using a LOD score of 20 (Lep-MAP3 modules SeparateChromosomes2 and OrderMarkers2). Resulting LGs with less than 100 markers were removed, resulting in 11 long, high-confidence LGs.

Next, we anchored the assembly scaffolds to the linkage map to create pseudomolecule sequences using Allmaps v0.9.14 (Tang et al. 2015). Again, gaps were filled with LR_Gapcloser and redundant scaffolds removed as above. The assembly was polished first using HyPo (downloaded February 23, 2020) (Kundu et al. 2019), with 10XG reads mapped by EMA and raw PacBio and ONT reads with Minimap2 v 2.17-r941 (Li 2018, 2). A second round of polishing was performed using EMA mappings of the 10XG reads with Pilon (Walker et al. 2014).

The mitochondrial genome was identified in the assembly by GC content and read coverage. The circular chromosome had been assembled into one contig, with a long overhang aligning back to itself. The ribosomal RNA large subunit (*rnl*) was identified in this contig by BLAST, using the *rnl* gene from the related species *Moniliophthora perniciosa* (NCBI GenBank: AY376688.1) as query. The contig representing the mitochondrial genome was broken manually within Geneious v10.2.4 (http://www.geneious.com), at a coordinate just before the *rnl* BLAST hit. The two resulting sequences were reassembled with the de novo assembly program in Geneious where the overlap was collapsed.

Genome completeness was assessed with BUSCO v2.0.1 with the basidiomycota_odb9 database (Simão et al. 2015) and telomeres were located using a custom script (available at https://github.com/markhilt/genome_analysis_tools/blob/master/find_telomeres.py). An assessment of bacterial contamination was performed using Blobtools v1.1.1 (Laetsch and Blaxter 2017), with the raw assembly as output by Canu, 10XG mapped reads (10X Genomics Longranger), and BlastN results to the nt database (1e-25) as input data.

From the single-spore sequencing data set, we calculated linkage disequilibrium between variants the following way. Reads were remapped to the finished assembly by BWA mem (Li and Durbin 2009) and variants were called using Platypus v0.8.1.1 (Rimmer et al. 2014). Heterozygous calls were disregarded and the resulting vcf file was recoded into a phased format (genotype calls are per definition phased as the spore data set is haploid). Linkage disequilibrium between variants on the same chromosome was calculated using the hap-r2 module of VCFtools v0.1.17 (Danecek et al. 2011).

Genome annotation was performed using Funannotate v1.8.3 (Palmer and Stajich 2021). We followed the Funannotate manual (https://funannotate.readthedocs.io; last accessed March 2021) closely during most steps, with the following exceptions. RepeatMasker v. open-4.0.9 (http://www.repeatmasker.org/RepeatMasker/) was run standalone to softmask the genome prior to annotation, using a custom, manually curated repeat library (described below). GeneMark-ET v4.62_lic (Lukashin and Borodovsky 1998) was included during structural gene predictions. Functional information about gene predictions was collected with eggNOG mapper online (http://eggnog-mapper.embl.de/; February 2, 2021), standalone runs of InterProScan v5.30-69.0 (Jones et al. 2014), and the fungal version of antiSMASH v5.1.1 (Blin et al. 2019). Phobius (Käll et al. 2004) was run using the wrapper from Funannotate.

We compiled a repeat library for the new assembly the following way. RepeatModeler v2.0.1 (Zeng et al. 2018) with the –LTRStruct option was initially used to mine the genome for repetitive sequences. Individual copies with flanking regions of the sequences from RepeatModeler were collected from the genome using BlastN, aligned with MAFFT v7.407 (Katoh and Standley 2013), manually inspected, and curated. We focused primarily on three points during manual curation: extending flanks to capture the whole element, creating a new consensus, and classifying the consensus sequence. Boundaries of each element were defined based on target site duplications (TSD), and for the new consensus, all sequence between the TSDs was included (but not the TSDs themselves). Structural information (e.g., LTR and TIR regions) was collected by self-alignments using MAFFT and used for classification, in addition to protein domains as identified by CDD (https://www.ncbi.nlm.nih.gov/Structure/cdd/wrpsb.cgi; last accessed February 15, 2021). We based our classifications on the system proposed by Wicker et al. (2007), following the structural information, TSD and protein domain architecture provided therein for defining *Gypsy*, *Copia*, and most DNA TEs. Additionally, *Plavaka* autonomous elements were defined by the pfam18759 domain, and *Dileera* and *Kyakuja* elements by protein homology of the transposase to previously identified families (Iyer et al. 2014). *Helitrons* were classified by having the pfam14214 and pfam05970 domains (Castanera et al. 2014). For LINEs we used homology to sequences in RepBase for classification, as identified by Censor (Bao et al. 2015). Nonautonomous elements were defined by having TSDs and structural features but lacking the protein domains required for transposition, also in most cases spanning less than 1,000 bp. We found only nonautonomous DNA elements, which we classified based on terminal sequence motifs and TSD length (Kojima 2020b; Storer et al. 2021). In cases where we had already identified autonomous families of certain elements, we used their TIR and TSD characteristics for classification of nonautonomous families. We noticed widespread insertions of putative nonautonomous MULE and *Academ* elements (Kojima 2020a). Reasoning that these superfamilies likely have autonomous families in the genome that had escaped capture by RepeatModeler, we used hmmer v3.3.2 (hmmer.org) to search for them. We used the hmm profile for pfam10551 for MULE, and for *Academ* we obtained an hmm profile from Muszewska et al. (2017). Resulting hits were curated the same way as above. We also searched for previously unidentified *Sola, Dada, Zator*, and *Kolobok* elements using hmm profiles from Muszewska et al. (2017) but without finding multiple well-supported hits.

## Supplementary Material

Supplementary data are available at *Genome Biology and Evolution* online.

## Supplementary Material

evab126_Supplementary_Data
