# Supplementary material for: The Assembled and Annotated Genome of the Fairy-Ring Fungus Marasmius oreades
Source: Genome Biol Evol. 2021 May 29;13(7):evab126. doi: 10.1093/gbe/evab126 (PMC8290104; doi:10.1093/gbe/evab126)
Supplement: evab126_Supplementary_Data [file evab126_supplementary_data.zip › Supplementary_figures.pdf]

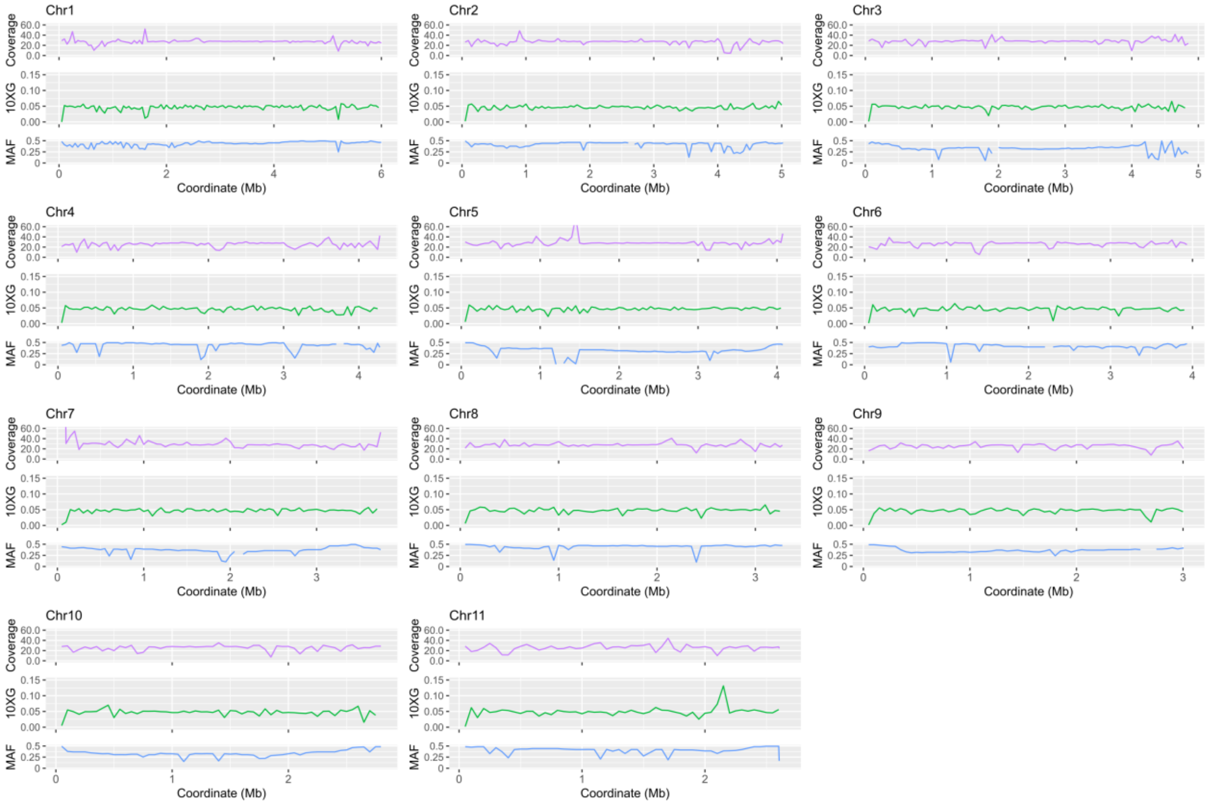

Supplementary Figure S1. Genome statistics. Panels from top to bottom: read coverage per base pair, median of 95 single-spore isolates; 10X Genomics Chromium barcode similarity (calculated as the shared number of barcodes between two windows, divided by the total number of barcodes in both windows); frequency of the minor allele in 95 single-spore isolates, median per window (variants overlapping with repetitive regions filtered out). The 10X barcode fraction is expected to be stable across well-assembled genomes. Windows are 50 kb in length and non-overlapping. Chr7 has a peak in coverage near the scaffold beginning (data point outside the y-axis limit). This peak corresponds to the rDNA cluster, which is highly repetitive and collapsed during assembly, leading to high read coverage.

A

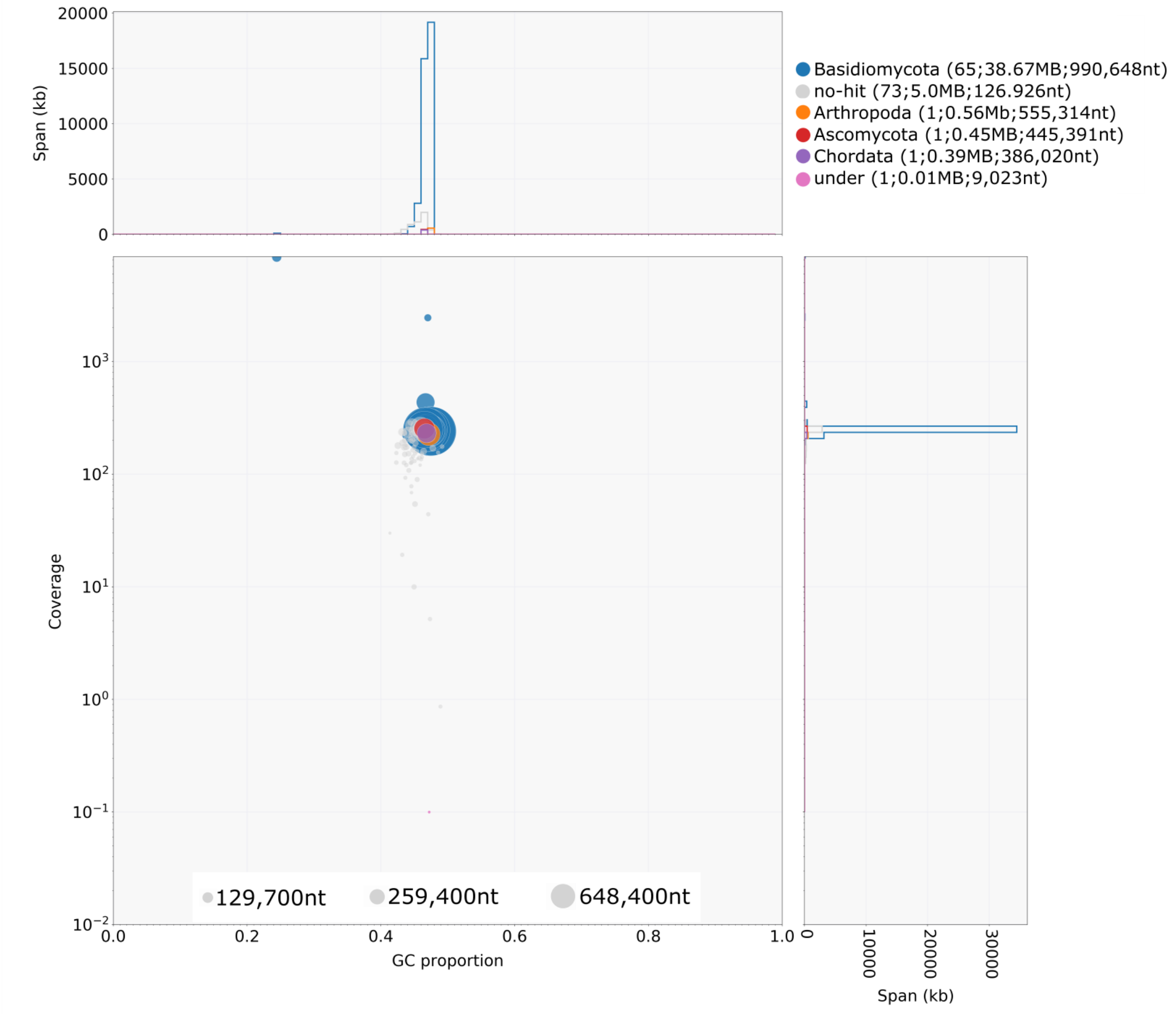

B

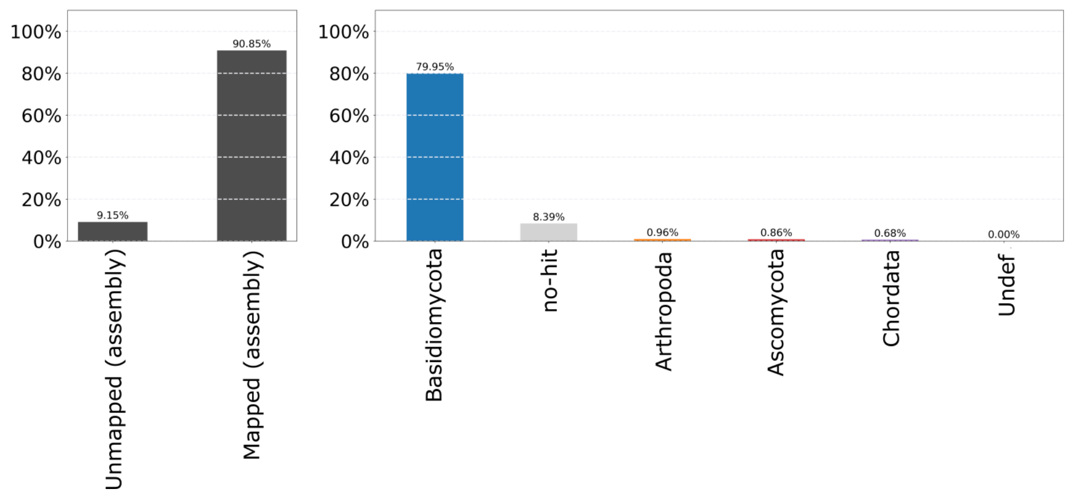

Supplementary Figure S2. Contamination assessment. A. Taxonomic assignment, average read coverage and GC content for each contig in the raw Canu assembly, as output by Blobtools. B. Percentage of 10X Chromium reads that mapped to the raw assembly (first panel) and taxonomic assignment of the contigs they mapped to (second panel).

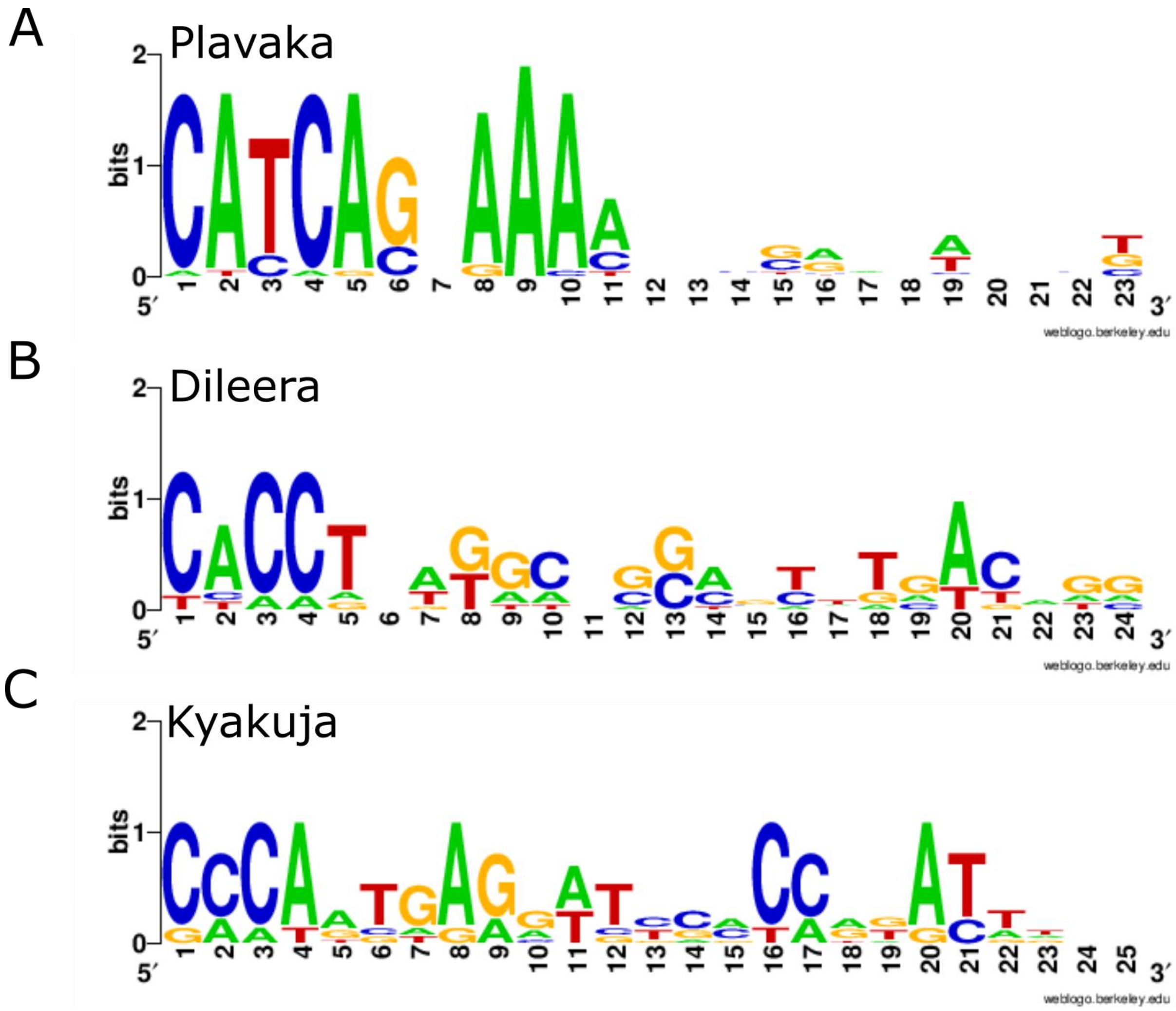

Supplementary Figure S3. Terminal inverted repeat sequence logos. A. *Plavaka*, B. *Dileera* and C. *Kyakuja* superfamilies of DNA transposons. Alignments were made from the consensus sequences of each element class (i.e. representatives in the repeat library), and letter size is proportional to occurrence of the base in the alignment.
